# Low selection rate of HLA-anchor escape mutations in HIV after transmission of subtype B and recombinants BF strains patients from Argentina

**DOI:** 10.1186/1742-4690-9-S2-P140

**Published:** 2012-09-13

**Authors:** G Damilano, E Socias, C Magneres, G Turk, M Gomez-Carrillo, H Salomon, D Dilernia

**Affiliations:** 1 Argentinean Reference Center for AIDS, Buenos Aires, Argentina; 2 HUESPED Foundation, Buenos Aires, Argentina

## Background

The immune response of HIV-infected individuals shapes the evolution of the virus by selecting escape mutation. After transmission to a new host, the HLA-mediated immune pressure changes. The objective of our study was to characterize the dynamics of HLA-anchor escape mutations after transmission.

## Methods

We studied 6 transmission events between members of serodiscordant couples collecting blood samples from the donor and the previously seronegative-partner at the moment of seroconversion, and a second sample at least 6 months post-infection. HLA-I typing was performed by the SSOP-PCR method. The viral gene gag was amplified by RT-PCR and cloned into the pGEM-T vector for viral quasiespecies analysis. Transmitted strain was identified by phylogenetic analysis. Escape mutation was defined as viral polimorphisms located in HLA-anchor position that eliminate an epitope predicted by the NetMHC (CBS Prediction Server). Significant variations in the number of escape mutations were assessed by Poisson´s probability distribution.

## Results

Of the total epitopes available in transmitted gag for recognition by the recipient HLA alleles, a mean 7.25% of them selected escape mutations by the moment of the second sample collection in an anchor position of the epítope. Of the total of HLA-anchor escape mutations present in the second sample and absent in the subtype consensus, a mean 79,8% were already present in the first sample of the recipient and in the sample of the donors. Also, the majority (84%) of the escape mutations to the donor’s HLA alleles persisted in time after transmission without reversion, even in the absence of the selecting HLA allele.

## Conclusion

The low rate of newly selected escape mutations could be due in part to lack of immune pressure, but considering the high rate of transmission and persistence, our results suggest a high level of viral adaptation to the HLA system in subtype B and recombinants BF circulating in Argentina.

